# Supplementary material for: The right thalamus may play an important role in anesthesia-awakening regulation in frogs
Source: PeerJ. 2018 Mar 15;6:e4516. doi: 10.7717/peerj.4516 (PMC5857353; doi:10.7717/peerj.4516)
Supplement: Figure S1 — There were 2 electrodes above the telencephalon, diencephalon and mesencephalon respectively, while the reference was above the cerebellum. [file peerj-06-4516-s003.doc]

**The Right Thalamus may Play an Important Role in Anaesthesia-Awakening Regulation in Frogs**

**Yanzhu Fan ^1,2^, Xizi Yue^1^,** **Fei Xue^1^, Steven E. Brauth^3^,** **Yezhong Tang^1^, and Guangzhan Fang^1,*^**

^1^Chengdu Institute of Biology, Chinese Academy of Sciences, No.9 Section 4, Renmin South Road, Chengdu, Sichuan, People's Republic of China

^2^University of Chinese Academy of Sciences, 19A Yuquan Road, Beijing, People's Republic of China

^3^Department of Psychology, University of Maryland, College Park, MD20742, USA

Email: [fanggz@cib.ac.cn](mailto:fanggz@cib.ac.cn)

**Supplementary figure**

**
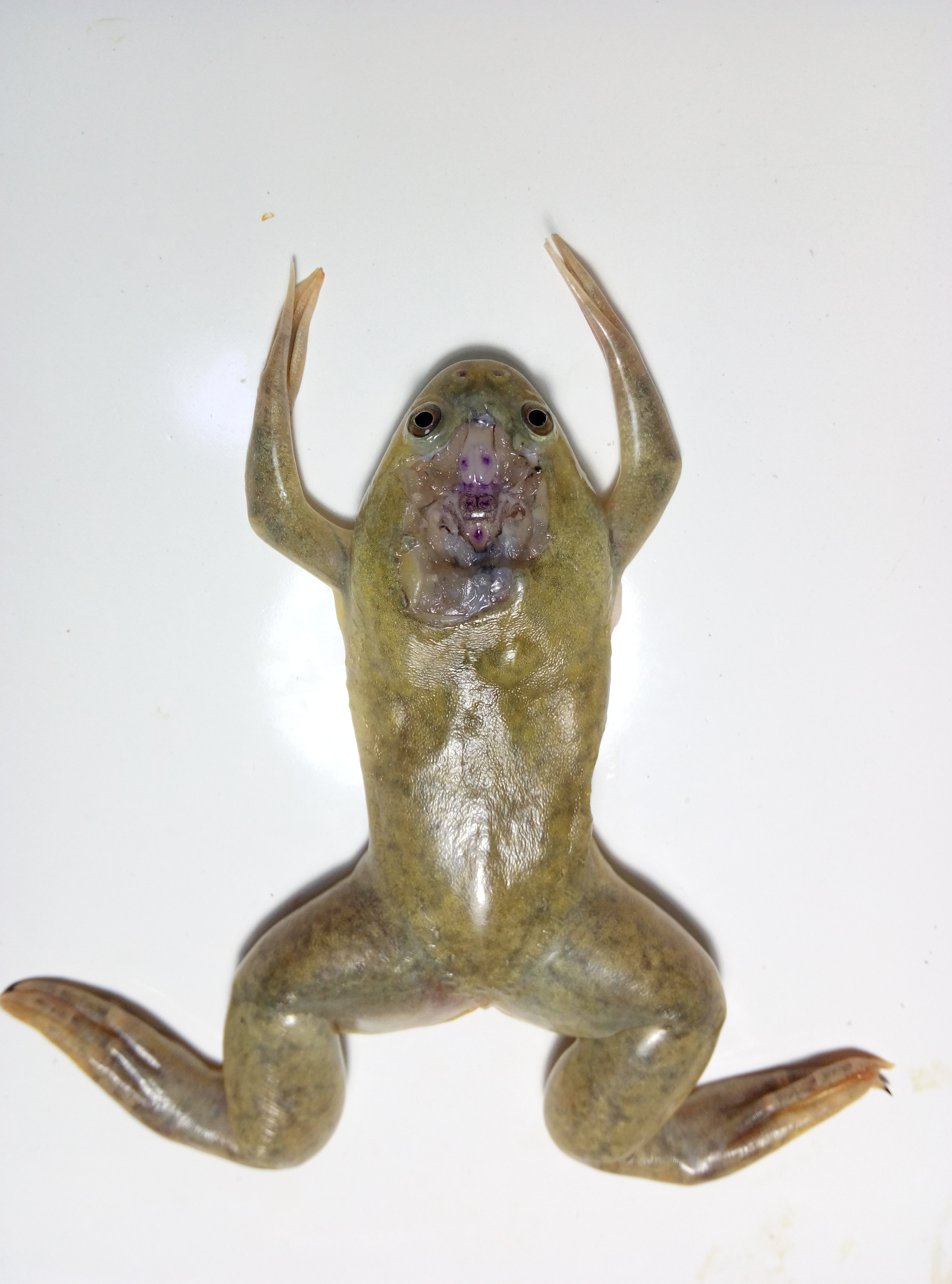
**

**Figure S1. An example showing the positions of 7 electrodes on the skull of a subject. There were 2 electrodes above the telencephalon, diencephalon and mesencephalon respectively, while the reference was above the cerebellum.**
